# Phytogenic Feed Additives as an Alternative to Antibiotic Growth Promoters in Broiler Chickens

**DOI:** 10.3389/fvets.2015.00021

**Published:** 2015-08-03

**Authors:** Ganapathi Raj Murugesan, Basharat Syed, Sudipto Haldar, Chasity Pender

**Affiliations:** ^1^BIOMIN America Inc., San Antonio, TX, USA; ^2^BIOMIN Holding GmbH, Getzersdorf, Austria; ^3^Department of Animal Nutrition, West Bengal University of Animal and Fishery Sciences, Kolkata, India

**Keywords:** digestibility, histomorphology, microbiota, performance, poultry

## Abstract

The recent trend toward reduction of antibiotic growth promoters (AGP) in North American poultry diets has put tremendous pressure on the industry to look for viable alternatives. In this context, phytogenic feed additives (PFA) are researched to improve gut health and thereby performance. An experiment was conducted with the objective to evaluate the effects of PFA as an alternative to AGP on small intestinal histomorphology, cecal microbiota composition, nutrient digestibility, and growth performance in broiler chickens. A total of 432-day-old Vencobb 400 broiler chicks were randomly assigned to one of three dietary groups, each consisting of 12 replicate pens (*n* = 12 chicks/pen). The chicks were fed a corn–soybean meal-based control (CON), CON + 500 mg/kg of AGP (bacitracin methylene disalicylate containing 450 mg active BMD/g), or CON + 150 mg/kg of proprietary blend of PFA (Digestarom^®^ Poultry) until 39 days of age when samples were collected. Birds fed either AGP or PFA had increased villus height in all three segments of the small intestine in comparison to the birds fed CON (*P* ≤ 0.05). Furthermore, the PFA-fed birds had significantly increased villus height and lower crypt depth compared to AGP fed birds (*P* ≤ 0.05). Birds fed either additive also had increased total tract digestibility of dry matter, crude protein, and ether extract (*P* ≤ 0.05). The strong effect of the PFA on villus height in the jejunum may suggest augmented nutrient absorption in PFA-fed birds. Although both additives reduced total cecal counts of anaerobic bacteria and *Clostridium* spp., PFA alone reduced the total coliform count while increasing the *Lactobacillus* spp. count (*P* ≤ 0.05). These results suggest the establishment of beneficial microbial colonies in PFA-fed birds. Overall, both PFA and AGP increased body weight gain while lowering the feed conversion ratio (*P* ≤ 0.05). Hence data from this experiment demonstrate the efficacy of PFA as a substitute to AGP in poultry diets.

## Introduction

Sustaining a healthy gut environment is a prerequisite to efficient broiler performance. Antibiotic growth promoters (AGP) have been used since the mid-1940s to maintain a healthy gut environment and in turn improve the performance (1). The ban on AGP compounds from poultry diets in Europe (2) and recent moves toward reduction or termination of AGP in North America have put pressure on the poultry industry to look for viable alternatives that can improve performance, protect animal health, and maintain profit margins (3). Phytogenic feed additives (PFA) have been gaining considerable interest lately due to their ability to improve performance by sustaining a healthy gut environment.

According to European Union legislation (EC 1831/2003) (4), PFA are categorized as sensory and flavoring compounds, which consist mainly of plant extracts (essential oils, oleoresins, and flavonoids) and their active principles (5). The essential oils present in PFA, which contain most of the active substances of the plant, have been suggested to increase the growth performance (6, 7), nutrient digestibility (8), and gut health (6, 9) in poultry species. The numerous beneficial qualities of PFA are predominantly derived from their bioactive molecules including carvacrol, thymol, capsaicin, cineole, etc. (10). It is these properties of PFA that project them as suitable alternatives to AGP.

The primary mode of action of PFA is controlling potential pathogens and beneficially modulating the intestinal microbiota. Several plant extracts are known to have antimicrobial, antiviral, anticoccidial, fungicidal, and/or antioxidant properties (11). Studies conducted in broilers have demonstrated the antimicrobial efficacy of PFA against pathogenic bacteria, such as *Escherichia coli* and *Clostridium perfringens*, potentially indicating a reduced risk for the development of colibacillosis and necrotic enteritis (8, 12). Alleviation of coccidiosis symptoms, including reduction in lesion severity and oocyst shedding, by PFA has also been documented (6). The antimicrobial effects of PFA are primarily attributed to their phenolic components and their action on pathogenic cells (13, 14).

It has been suggested that PFA augment nutrient utilization in the gastrointestinal tract (GIT) by enhancing production of digestive secretions and enzymatic activity (11, 15). Furthermore, several studies have observed positive effects of PFA on the morphology of small intestinal tissues, such as increased villus height, decreased crypt depth, and increased goblet cell counts (16–18). Such effects on gastrointestinal morphology have been postulated to increase the nutrient digestibility in poultry (19). PFA, like AGP, may also reduce mucosal thickness, thus contributing to the diffusion of nutrients to the apical surface of epithelial cells and increased absorption and feed efficiency (20).

Overall, PFA are capable of reducing microbial threat and promoting intestinal health, which is imperative for optimal bird performance and profitability. The objective of this study was to evaluate the efficacy of PFA as an alternative to AGP in broiler production by determining their effects on cecal microbiota composition, small intestinal histomorphology, nutrient digestibility, and growth performance.

## Materials and Methods

### Animals and housing

All animal procedures were conducted according to the ethical norm of West Bengal University of Animal and Fishery Sciences. An experiment of 39 days duration was conducted using a total of 432-day-old Vencobb 400 broiler chicks, which were randomly assigned to one of three dietary groups at the start of experiment. The 3 groups consisted of 12 replicate pens of 12 chicks, each resulting in 144 birds per group.

### Diets and groups

The dietary groups were (i) control (CON), (ii) basal diet + AGP (AGP), and (iii) basal diet + PFA (PFA). The CON diet consisted of a corn–soybean meal-based basal diet, and was formulated to meet or exceed breeder recommendations [Table 1; “Nutrient Levels,” Venky’s Ltd. (21)]. The AGP used was bacitracin methylene disalicylate containing 450 mg active BMD/g at the inclusion rate of 500 mg/kg of diet. The PFA used was Digestarom^®^ Poultry (Biomin Holding GmbH, Austria) at the inclusion rate of 150 mg/kg of diet. The PFA contains a combination of over 30 essential oils and phytogenic compounds. Neither exogenous polysaccharide-degrading enzymes nor anticoccidial drugs were added in the diets as these might partly mask the effects of the PFA, but a mycotoxin binder was used.

**Table 1:** **Formulation and composition (gram/kilogram) of the experimental diets**.

	Starter (1–7 days)	Grower (8–21 days)	Finisher (22–39 days)
Ingredients			
Ground corn	540.7	567.2	583.4
Soybean meal (46% CP)	396.0	362.5	328.0
Soybean oil	27.0	33.7	51.1
Calcite powder	12.45	12.45	12.45
Di-calcium phosphate	16.5	16.5	17.0
D-methionine	0.55	0.95	1.35
Lysine hydrochloride	0.3	0.2	0.2
Sodium bicarbonate	2	2	2
Salt	2	2	2
Premix^a^	2	2	2
Toxin binder	0.05	0.5	0.5
Calculated composition (g/kg as-fed)			
ME_N_ (MJ/kg)	11.85	12.14	12.65
Crude protein	223.5	210.4	196.3
Ether extract	52.7	59.8	7.33
Crude fiber	2.5	2.4	2.4
Calcium	9.57	9.47	9.38
Available P	3.18	3.16	3.13
Digestible lysine	11.02	10.19	9.39
Digestible methionine	3.51	3.76	3.99
Digestible methionine + cysteine	6.87	6.73	6.77

*^a^Including vitamins and trace minerals*.

### Measurements of production traits

Birds were monitored twice daily and any mortality was removed, weighed, and recorded. Birds were individually weighed at 7, 21, and 39 days, which designated the end of pre-starter, starter, and grower periods, respectively. Total feed intake (FI) per pen was also measured on the same days. Body weight gain (BWG), average daily feed intake (ADFI), and feed conversion rate (FCR) were calculated per pen for each period and for the overall period. Mortality BWG data were used to correct the FCR.

### Determination of apparent total tract nutrient digestibility

Two birds were randomly selected from each pen and were distributed to cages at 30 days of age (eight cages per group, three birds per cage) to provide an acclimatization period of 6 days. Total excreta collection was done for three consecutive days from 36 to 39 days of age at 2 h intervals. Samples were mixed, weighed, and a 10% aliquot was frozen at −20°C. The sampled excreta were again pooled by cage at the end of the collection period, and a 10% aliquot was taken and dried. Feed samples were collected daily for the same 3-day period (36–39 days) and pooled to produce a single composite of each diet. Diet and excreta samples were analyzed to determine the apparent total tract digestibility of dry matter (DM), crude protein (CP), and ether extract (EE) (22, 23).

### Histomorphology of the small intestine

One bird per replicate pen was euthanized via carbon dioxide asphyxiation at the end of the experiment on day 39. The entire GIT tract was removed aseptically before separating into sections of duodenum, jejunum, ileum, cecum, and colon. The small intestinal segments (duodenum, jejunum, and ileum) were processed for histomorphological analysis (24). Segments measuring 2-cm in length from the mid-points of the duodenum, jejunum, and ileum were cut, flushed with cold saline, fixed in 10% buffered formalin, and stained with hematoxylin–eosin. Histological sections were examined with a phase contrast microscope coupled with a deconvolution imaging analysis system [VayTek^®^, Fairfield, IA, USA; (25)]. Villus height (VH, from the tip of the villus to the top of the lamina propria), crypt depth (CD, from the base to the region of transition between the crypt and villus), and the thickness of the muscularis mucosae in the duodenum, jejunum, and ileum were determined. Measurements of 10 complete villi for VH and associated crypts for CD were taken from each segment, and the average of these values was used for statistical analysis.

### Caecal microbiota composition

The ceca with contents were stored at 4°C for determination of cecal microbiota. The cecal digesta was processed within 24 h (5). Each cecal digesta homogenate was serially diluted from 10-1 to 10-8. Coliforms were cultured using HiCrome *E. coli* HiVeg Agar (Product code: MV1295; Hi-Media Laboratories, Mumbai, India), anaerobic bacteria were cultured using Wilkins Chalgren Anaerobic Agar (Product code: M832; Hi-Media Laboratories), *Staphylococcus aureus* was cultured using HiCrome Staph Agar (Product code: M1837; Hi-Media Laboratories), *Lactobacillus* spp. were cultured using *Lactobacillus* MRS Agar (Product code: M641; Hi-Media Laboratories), *Pseudomonas aeruginosa* was cultured using Cetrimide Agar (Product code: M024; Hi-Media Laboratories), and the *Clostridium* were cultured in Reinforced Clostridial Agar (Product code: M154; Hi-Media Laboratories). Diluted digesta samples were streaked onto the agar plates and incubated at 37°C for 48 h, while the clostridia plates were incubated anaerobically at similar temperature. Visible colonies were enumerated using a colony counter and the results were expressed as log_10_ CFU/g of cecal digesta.

### Statistical analysis

The data were analyzed as a complete randomized design using General Linear Model procedure of SPSS (version 17.0). Repeated measures ANOVA was used to analyze performance parameters, which were measured over the course of the experiment. Means were separated using Tukey-WSD following ANOVA. Values were considered statistically different at *P* ≤ 0.05. The replicate pens were used as experimental units for all parameters except apparent total tract nutrient digestibility where the cages were used as experimental units.

## Results

### Production performance

#### Body Weight Gain

Mortalities for the groups were statistically insignificant at 4.22, 2.22, and 2.78% for the CON, AGP, and PFA groups, respectively, for the 1–39 days period. No significant differences in BWG were noted during the 1–7 days pre-starter phase (Table 2). There was an effect of dietary treatment on BWG during the starter phase (8–21 days; *P* = 0.03), grower phase (22–39 days; *P* < 0.01), and during the overall period (1–39 days; *P* < 0.01). During the starter phase, birds-fed AGP had significantly increased BWG compared to CON-fed birds (*P* ≤ 0.05), while the BWG for PFA-fed birds was not significantly different from either CON or AGP groups. During the grower phase, birds-fed PFA had significantly increased BWG in comparison to both CON (*P* ≤ 0.05) and AGP groups (*P* ≤ 0.05), which did not differ from each other. Supplementation of either AGP (*P* ≤ 0.05) or PFA (*P* ≤ 0.05) to the basal diet significantly increased BWG for the overall experimental period.

**Table 2:** Performance of broilers supplemented with either an antibiotic or a phytogenic feed additive during 1–39 days of age*.

	Dietary treatments^†^			SEM	*P* value
	Control	AGP	PFA	
Pre-starter period (1–7 days)					
Body weight gain (g)	140.5	144.3	142.3	1.20	0.44
Feed intake (g)	185.4	185.9	185.5	1.03	0.98
Feed conversion^‡^	1.323	1.292	1.305	0.012	0.57
Starter period (8–21 days)					
Body weight gain (g)	823.4^b^	860.3^a^	835.1b^ab^	5.93	0.03
Feed intake (g)	1004.5^a^	1007.8^a^	975.3^b^	4.96	0.01
Feed conversion^‡^	1.221^a^	1.173b^ab^	1.169^b^	0.089	0.02
Grower period (22–39 days)					
Body weight gain (g)	1073.5^b^	1109.2^b^	1183.1^b^	12.49	<0.01
Feed intake (g)	2599.6	2606.5	2590.6	7.56	0.71
Feed conversion^‡^	2.431^a^	2.355^a^	2.195^b^	0.028	<0.01
Overall period (1–39 days)					
Body weight gain (g)	1896.9^b^	1969.5^a^	2018.2^a^	14.41	<0.01
Feed intake (g)	3789.5	3800.2	3751.5	9.81	0.10
Feed conversion^‡^	2.002^a^	1.931b^ab^	1.860^b^	0.015	<0.01

*Means with dissimilar letters in a row varied significantly*.

*Means of 12 replicate pens (*n* = 12 birds per pen).

*^†^Supplemented with either an antibiotic growth promoter, bacitracin methylene disalicylate, 500 mg/kg (AGP), or a phytogenic feed additive (Digestarom^®^ Poultry) 150 mg/kg (PFA). The control group received the unsupplemented basal diet*.

*^‡^Corrected for mortality*.

#### Feed Intake

No significant differences in FI were noted among the groups throughout the experimental period, except during the starter phase (8–21 days; *P* = 0.01) when the FI of PFA-fed birds was significantly lower in comparison to both AGP- (*P* ≤ 0.05) and CON-fed birds (*P* ≤ 0.05; Table 2).

#### Mortality-Corrected Feed Conversion Ratio

No significant differences in FCR were present during the 1–7 days pre-starter phase (Table 2). There was an effect of treatment on FCR during the 8–21 days starter phase (*P* = 0.02), 22–39 days grower phase (*P* < 0.01), and for the overall period (1–39 days; *P* < 0.01). Supplementation of PFA lowered the FCR in comparison to CON-fed birds during all time periods (*P* ≤ 0.05). The FCR for birds-fed AGP did not differ significantly from either the CON or PFA groups for the starter phase as well as for the overall period. During the grower phase, however, PFA-fed birds had significantly lower FCR in comparison to the AGP-fed group (*P* ≤ 0.05).

### Nutrient digestibility

There was a significant effect of dietary treatment on apparent total tract digestibility of DM (*P* < 0.01), CP (*P* = 0.04), and EE (*P* = 0.02; Table 3). The apparent total tract DM digestibility for the PFA-fed group was increased in comparison to the CON group (*P* ≤ 0.05), while the AGP group was not different from either the CON or PFA groups. Supplementation of either AGP or PFA to the basal diet significantly increased the apparent total tract digestibility of CP and EE when compared to the CON group (*P* ≤ 0.05).

**Table 3:** Nutrient digestibility of broilers supplemented with either an antibiotic or a phytogenic feed additive during 1–39 days of age*.

	Dietary treatments^†^			SEM	*P* value
	Control	AGP	PFA	
Nutrient digestibility (g/g intake)					
Dry matter	0.674^b^	0.711b^ab^	0.744^a^	0.007	<0.01
Crude protein	0.761^b^	0.784^a^	0.794^a^	0.005	0.04
Ether extract	0.736^b^	0.781^a^	0.782^a^	0.005	0.02

*Means with dissimilar letters in a row varied significantly*.

*Digestibility trial was conducted at 36 days of age for three consecutive days. Randomly selected birds were placed in metabolism cages. There were eight cages per treatment with three birds in each cage.

*^†^Supplemented with either an antibiotic growth promoter, bacitracin methylene disalicylate, 500 mg/kg (AGP), or a phytogenic feed additive (Digestarom^®^ Poultry) 150 mg/kg (PFA). The control group received the unsupplemented basal diet*.

### Histomorphology of small intestine

Dietary treatment had a significant effect on VH in the duodenum (*P* = 0.02), jejunum (*P* < 0.01), and ileum (*P* < 0.01; Table 4). The VH was significantly increased by AGP supplementation in the duodenum in comparison to the CON group (*P* ≤ 0.05), while PFA was not different from either AGP or CON. In the jejunum, both AGP and PFA significantly increased the VH in comparison to CON (*P* ≤ 0.05); however, the PFA group had significantly increased VH compared to AGP-fed birds (*P* ≤ 0.05). Additionally, both AGP and PFA significantly increased the ileal VH in comparison to the CON group (*P* ≤ 0.05). Overall, the supplementation of either AGP or PFA increased the VH across the small intestine.

**Table 4:** Villus height, crypt depth, and thickness of muscularis mucosae (micrometer) of broilers supplemented with either an antibiotic or a phytogenic feed additive during 1–39 days of age*.

	Dietary treatments^†^			SEM	*P* value
	Control	AGP	PFA	
Duodenum					
Villus height	2549.1^b^	3481.1^a^	2903.4b^ab^	140.23	0.02
Crypt depth	45.3	42.7	32.8	2.55	0.10
Mucosa thickness	387.1^a^	183.9^b^	230.4^b^	22.69	<0.01
Jejunum					
Villus height	2583.6^c^	2969.9^b^	3290.1^a^	280.51	<0.01
Crypt depth	29.8^a^	31.1^a^	20.2^b^	1.31	<0.01
Mucosa thickness	206.8	215.6	212.9	6.85	0.88
Ileum					
Villus height	2050.1^b^	2736.4^a^	2839.9^a^	94.03	<0.01
Crypt depth	34.1	30.9	31.6	1.04	0.45
Mucosa thickness	320.3^a^	233.9^b^	211.8^b^	14.31	<0.01

*Means with dissimilar letters in a row varied significantly*.

*Means of 12 birds per treatment. Birds were randomly selected and euthanized at 39 days of age.

*^†^Supplemented with either an antibiotic growth promoter, bacitracin methylene disalicylate, 500 mg/kg (AGP), or a phytogenic feed additive (Digestarom^®^ Poultry) 150 mg/kg (PFA). The control group received the unsupplemented basal diet*.

No significant differences in CD were noted in any dietary group in the duodenum or ileum (Table 4). There was a significant effect of dietary treatment in the jejunum (*P* < 0.01), where inclusion of PFA in the basal diet lowered the CD in the jejunum when compared to both CON and AGP groups (*P* ≤ 0.05).

Additionally, there was a significant effect of treatment on mucosal thickness in the duodenum (*P* < 0.01) and ileum (*P* < 0.01; Table 4). Both AGP and PFA significantly lowered the mucosal thickness in the duodenum and ileum in comparison to the CON group (*P* ≤ 0.05), but no differences were present in the jejunal mucosal thickness.

### Cecal microbiota composition

The composition of cecal microbiota at 39 days of age in log_10_ CFU/g of wet cecal digesta is provided in Table 5. There was a significant effect of dietary treatment on total coliform (*P* < 0.01), anaerobic bacteria (*P* < 0.01), *Lactobacillus* spp. (*P* < 0.01), and *Clostridium* spp. (*P* = 0.01) counts. Supplementation of PFA significantly decreased the total coliform count in comparison to the CON and the AGP groups (*P* ≤ 0.05). The CFU of total anaerobic bacteria and total *Clostridium* were decreased with the supplementation of either AGP or PFA (*P* ≤ 0.05). Supplementation of either AGP or PFA tended to decrease *Staphylococcus aureus* in comparison to the CON group (*P* = 0.06). No effect of dietary treatment on the cecal population of *Pseudomonas aeruginosa* was noted; however, supplementation of PFA significantly increased the number of cecal *Lactobacillus* in comparison to the CON and AGP groups (*P* ≤ 0.05).

**Table 5:** Cecal microbiota composition (log_10_ CFU/g wet cecal digesta) of broilers supplemented with either an antibiotic or a phytogenic feed additive during 1–39 days of age*.

	Dietary treatments^†^			SEM	*P* value
	Control	AGP	PFA	
Coliforms	5.54^a^	5.62^a^	5.10^b^	0.07	<0.01
Anaerobic bacteria	5.91^a^	5.64^b^	5.53^b^	0.04	<0.01
*Staphylococcus aureus*	2.61	1.24	1.14	0.49	0.06
*Pseudomonas aeruginosa*	4.81	4.74	4.84	0.06	0.27
*Lactobacillus* spp.	4.96^b^	5.01^b^	5.35^a^	0.04	<0.01
*Clostridium* spp.	5.17^a^	4.95^b^	4.97^b^	0.03	0.01

*Means with dissimilar letters in a row varied significantly*.

*Means of 12 birds per treatment. Birds were randomly selected and euthanized at 39 days of age.

*^†^Supplemented with either an antibiotic growth promoter, bacitracin methylene disalicylate, 500 mg/kg (AGP), or a phytogenic feed additive (Digestarom^®^ Poultry) 150 mg/kg (PFA). The control group received the unsupplemented basal diet*.

## Discussion

Supplementation of PFA significantly lowered the overall FCR by 3.6% (7 points) and 7.0% (14 points) in comparison to the AGP and CON groups, respectively. This was in agreement with earlier reports (5), which indicated improvement in final BW and FCR with PFA supplementation without any effect on the FI. The growth promoting effects of the PFA and the AGP in this experiment may be correlated with the significant increase in apparent total tract digestibility of nutrients. Furthermore, a significant decrease in FI in the PFA group was noted during the starter phase, perhaps suggesting that the bird’s nutrient requirements are being satisfied with a smaller quantity of feed (26). It has been well documented that AGP-increased nutrient digestibility is mostly due to their effects on the intestinal microbiota (27). Increased nutrient digestibility with the addition of PFA and growth performance similar to Avilamycin was reported by Hernandez et al. (19). It has been noted that extracts from spices and herbs may stimulate digestive secretions and enzymatic activity, thus exerting beneficial actions within the digestive tract (28). The present findings further support the idea that PFA favorably modulate gut functions and digestive activities to stimulate growth in broilers.

Morphological changes in the GIT caused by PFA may provide further information on possible benefits to the digestive tract. In general, PFA and AGP significantly increased the VH across the small intestine. In the absence of any inflammation, this ought to increase absorptive surface area and efficiency of digestion and absorption (29). A similar effect of PFA on VH has been reported by Namkung et al. (17). The positive effect of PFA in the present experiment in increasing the VH in the jejunum could increase the efficiency of absorptive process considering the fact that the majority of absorption occurs in the jejunum. Furthermore, greater VH increases the activities of mucosal digestive enzymes, resulting in improved digestibility (30, 31). As intestinal crypts are the source of epithelial cells for villi and CD is directly correlated with epithelial cell turnover, the shallower crypts in the jejunum due to PFA supplementation may be indicative of decreased cellular turnover and improved intestinal health. Moreover, cellular turnover is an energy consuming process that uses resources that might otherwise be utilized toward growth; thus, shallower crypts are also related to improved performance (32). In the current study, mucosa thickness was significantly reduced in the duodenums and ileums of AGP and PFA supplemented birds. Gordon and Bruckner-Kardoss (33) reported that germ-free birds had thinner muscularis mucosa than the birds reared under conventional management systems, indicating that thinning of the mucosa might spare nutrients for productive processes and this was reflected in the BWG of the birds.

Literature depicting antimicrobial role of PFA is ample (8, 12, 14, 34, 35), while very few explore the mode of action by which the PFA may facilitate the proliferation of beneficial bacteria, such as *Lactobacillus* spp. (5, 8). In the present experiment, PFA significantly reduced the cecal population of coliforms and fortified the gut microbiota with beneficial bacteria, such as *Lactobacillus* spp. Once the *Lactobacillus* spp. are established, they might selectively exclude the pathogens from adhering due to their fast colonization, proliferation, and acidification properties in the GIT (9). The inhibitory effect of PFA on *Clostridium* spp. is encouraging and paves the way for removal of the AGP from poultry diets. The essential oils present in PFA have been shown to inhibit the growth of *Clostridia* (34). Mitsch et al. (12) opined that PFA stabilizes the gut microbiota and thereby reduces the colonization of *Clostridia* in gut. Evaluation of the cecal microbiota population in this study has revealed that AGP and PFA alike reduced total bacterial load in the gut. This inhibitory effect of PFA on bacterial load may alleviate pressure on the immune system, thus allowing the reallocation of energy toward improving performance. Overall from the gut perspective, the PFA purportedly favored a healthy gut, which in turn could be concomitant with the growth enhancement.

In conclusion, supplementation of either AGP or PFA increased the apparent total tract nutrient digestibility by increasing the VH throughout the small intestine. In comparison to the AGP, the PFA supported establishment of a favorable gut microbiota composed of higher numbers of *Lactobacillus* spp. and less *Clostridium* spp. Furthermore, supplementation of PFA to a corn–soybean meal-based coccidiostat free diet increased the BWG and lowered the FCR in 39 days, which was comparable to the AGP used in this experiment. Overall, the present work demonstrated the efficacy of PFA utilization and confirms the importance of considering the inclusion of PFA in poultry diets as an alternative to AGP.

## Author Note

Presented in the Gut Health Symposium held at St. Louis, MO, USA on November 12, 2014.

## Conflict of Interest Statement

The authors declare that the research was conducted in the absence of any commercial or financial relationships that could be construed as a potential conflict of interest.
