# Corrigendum: Phytogenic Feed Additives as an Alternative to Antibiotic Growth Promoters in Broiler Chickens

**DOI:** 10.3389/fvets.2015.00037

**Published:** 2015-09-22

**Authors:** Ganapathi Raj Murugesan, Basharat Syed, Sudipto Haldar, Chasity Pender

**Affiliations:** ^1^BIOMIN America Inc., San Antonio, TX, USA; ^2^BIOMIN Holding GmbH, Getzersdorf, Austria; ^3^Department of Animal Nutrition, West Bengal University of Animal and Fishery Sciences, Kolkata, India

**Keywords:** digestibility, histomorphology, microbiota, performance, poultry

There is a typographical error in Table 2 of this article. Under the “Grower period (22–39 days)” section, the body weight gain for the PFA group should have the superscript “a,” instead of “b,” indicating that it is significantly different from the Control and AGP groups.

## Conflict of Interest Statement

The authors declare that the research was conducted in the absence of any commercial or financial relationships that could be construed as a potential conflict of interest.

