# Corrigendum II: Phytogenic Feed Additives as an Alternative to Antibiotic Growth Promoters in Broiler Chickens

**DOI:** 10.3389/fvets.2016.00028

**Published:** 2016-04-04

**Authors:** Ganapathi Raj Murugesan, Basharat Syed, Sudipto Haldar, Chasity Pender

**Affiliations:** ^1^BIOMIN America Inc., San Antonio, TX, USA; ^2^BIOMIN Holding GmbH, Getzersdorf, Austria; ^3^Department of Animal Nutrition, West Bengal University of Animal and Fishery Sciences, Kolkata, India

**Keywords:** digestibility, histomorphology, microbiota, performance, poultry

## Revised Conflict of Interest Statement

The authors declare that MICRO-PLUS Konzentrate GmbH, now a subsidiary of BIOMIN Holding GmbH, financially sponsored this project. The authors GM and CP are affiliated with BIOMIN, which produces the phytogenic feed additive used in this trial. BS, affiliated with MICRO-PLUS Konzentrate GmbH at the time the experiment was conducted, is currently affiliated with BIOMIN following the acquisition.

## Ethics Declaration

At the time this experiment was conducted, the Institutional Animal Ethics Committee standards for conducting research on poultry species were not established, thus no committee approval was required, and the experiment was conducted according to the ethical norms of the University. The principal investigator of the study has provided the journal’s Editorial Office a retrospective statement that approval was not needed.

## Author Contributions

GM: final approval of the version to be submitted, drafting the article, or revising it critically for important intellectual content; BS: contributions to conception and design of the experiment and analysis and interpretation of data; SH: contributions to conception and design of the experiment, acquisition of data, and analysis and interpretation of data; CP: final approval of the version to be submitted, drafting the article, or revising it critically for important intellectual content.

